# The Hands

**Published:** 1889-02

**Authors:** M. C. Newhaus, E. Heron Allen


ARTICLE II.

### THE HANDS.

BY MRS. M. C. NEWHAUS.

[With remarks and quotations from M. D'Arpentigny, translated chiefly by E. Heron Allen.]

The hand is the member of all members. Its importance has been especially commented upon, by all writers, historians, and soothsayers since the world began. There is scarcely an event in our lives but the hand has been the principal agent. Its beauty is beyond description. What fine mechanism, what curious construction, and what wonderful responsiveness to the most delicate sensibility. The joy and sorrow, the tragedies and mysteries that emanate from it. Through it we live again with all the poets, philosophers, and scientists of old. The most dreaded and disgraceful punishment in olden times was the disfigurement or severing of the hand. Superstition has also run riot in regard to hands as the itching of the palm which was supposed to foretell wealth, etc. Heron Allen, in his last work, tells of some of the old superstitions about the hands and nails, and cutting of them. The The ancient dogma which we all know of "cutting on Monday you cut them for health, cutting on Tuesday cut them for wealth, etc.," and one and all agreed that "A man had better never been born, than have his nails on a Sunday shorn." The Romans performed this operation on a day especially set aside for the purpose.

We all naturally have a preference for small beautifully molded hands, and would be inclined to think they indicated great delicacy of mind and heart; but strange as it may seem, some of the most cruel and relentless characters in history possessed such hands. Charlotte Corday had beautiful delicate hands—which hardly suggested strength or courage enough to drive a dagger to the heart of Murat.

The importance and significancy of the hand is plainly seen all through life. Slander can be overlooked, rudeness ignored, but the raising of the hand against another is an insult beyond forgiveness. The ancient Persians used to keep their hands folded beneath their robes when in the presence of their ruler as a sign of absolute submission. What greater mark of humility than an appeal made with clasped hands!

A maiden in "giving her hand" yields up her liberty.

D'Arpentigny writes "That there are hands which naturally attract us, and there are hands which excite in us repulsion. I have seen hands that seemed covered with *eyes*, so sagacious, so penetrating was their appearance. Some, like those of the sphynx, suggest an idea of mystery, some betray folly and strength combined with activity of body; others again indicate laziness joined to feebleness and cunning." And again he writes "Large hands, particularly if they be hard, are a sign of physical strength, and as the Greeks could not conceive beauty without strength, a large hand was to them a great beauty. It is a matter of common knowledge that the Greeks, whatever their station of life, never rode when they might walk, did their own cooking, and otherwise performed a great deal of manual labor which to-day would be looked upon with aversion and contempt." Large hands have an inherent love of detail, and is its especial attribute. Now, little hands, on the contrary, affect not only the large, but the colossal. The pyramids and temples of Egypt and India have all been built by people with the most delicate hands in the world.

In an article on Palmistry written some months ago I briefly enumerated the types of hands without any special analysis, as the work dwelt more particularly upon the lines of the palm. Nearly every one in asking me to examine their hands ask, "How can I tell if my hand is larger or smaller than that of another? How is the expression any different than my neighbor's." It would seem difficult to answer, and one can only distinguish these curious differences by constant study of all hands that present themselves. There is nothing that indicates character any more distinctly than the hand, to a student of this science. Like the human face, we never find the hands of two persons exactly alike. D'Arpentigny gives seven classes or types of hands.

1. The Elementary, or Large-Palmed Hand.
2. The Necessary, or Spatulated Hand.
3. The Artistic, or Conical Hand.
4. The Useful, or Square Hand,
5. The Philosophic, or Knotty Hand.
6. The Psychic, or Pointed Hand.
7. The Mixed Hand.

The Elementary hands are large and thick with little suppleness and hard in texture, and belong almost entirely to those who live by manual labor and whose lives are governed by routine. They are heavy and sluggish, with dull imagination—yet the influence of these hands is unquestionable, especially in our own country where the farmer lays the foundation of our wealth. Just a moment's thought will bring to our minds the lovely gardens and parks, grand boulevards and walks which these heavy and clumsily handed people perfect so beautifully. Superstition is strongly marked in this type and wizards and spectres are often supposed to be seen by these persons. Strong and powerful as is this type it is a strange thing that when illness attacks them they succumb readily, from the fact no doubt that they are lacking in resources and strength of will.

The Spatulated hand, by this term we mean hands which present a flattened out appearance at the end and resemble a *spatula*. This hand indicates resolution, energy, resource for resisting physical ills, love of life and strong intelligence. All the greater workers, explorers, navigators, were of this type. The fingers are smooth and indicate love of comfort and elegance, of a material, rather than of an artistic kind. The Scotch and English greatly favor this hand, but in Russia it is more commonly found. Persons with spatulated hands are more Platonic than personal in their love, more prone to Protestantism than Catholicism, because the Catholic appreciates, to a higher degree, the *sentiment* of religion, but the *idea* of religion is more highly developed among the Protestants, as the spatulated hand is more material than spiritual, more practical than sentimental. Englishmen who very commonly possess those hands are adroitly commented upon by Bulmer Lytton, who writes of these people perhaps a little severely, but in the main he is correct.

"The English make business an enjoyment and enjoyment a business; they are born without a smile; they move about in public places like so many easterly winds—cold, sharp, and cutting; or like a group of fogs on a frosty day, sent out of his hall by Boreas for the express purpose of *looking black at one another.* When they ask you 'How do you do?' you would think they were measuring the length of your coffin. They are even, it is true, *laboring to be agreeable*; but they are like Sisyphus. The stone they roll up the hill with so much toil runs down again, and hits you a thump. They are sometimes polite, but invariably uncivil. Their warmth is always artificial, their cold, never, they are stiff without dignity. They offer you an affront, and call it "a plain truth," they wound your feeling and tell you its manly "to speak their minds," while they profess to abhor servility, they adulate the peerage." Henrich Heine, the great German poet and cynic; possessed very delicate hands, greatly in contrast to the practical spatulated hands of the English, whose coolness and lack of sentiment made him antagonistic—he writes as follows in his Pictures of Travel:

"Kind Nature rarely disinherits her creatures. While she has denied the English the profession of much that is beautiful, and lovely, has given them neither voice for song nor taste for real enjoyment, and has endowed them with leather ale pouches, instead of human souls. She has given them in compensation, a large share of civil liberty, the art of comfortably arraying their homes, and William Shakespeare,"

But of this type is much good to be said, some of the most wonderful, and powerful manufactoriss in the world are the work of these hands. In America we find a great variety of types—as we are a people of many nations.

The Conic or artistic type is modified in its formation, having three different characteristics. Supple, with small thumb, showing love of beaucy and form; large, with thumb thick and short, it seeks power and wealth. Napoleon had a hand of this kind. The last variation in this type has a large palm and thumb while the texture is hard, showing great tendency to fatalism. *All* these variations are governed by inspiration, and are lacking in mechanical arts. The French soldiers who are enthusiastic and emotional and are moved by eloquence, have the Conic or artistic hands. The phlegmatic German soldiers show more of the elementary type. The English the spatulated. To the latter *war is a trade*, a profession, where pay is its object. The Americans where *all* types are represented fight with the endurance, enthusiasm, and courage of all. The love of persons with artistic hands is more sensual than that of the square or spatulate types. There is also a lack of order and prudence Spatulate handed persons view things as they are, the Conic artistic as they *imagine them to be.*

The Square or useful hand. It is of medium size, knotty fingers, square tips, large thumb with ball well developed, palm hollow and texture firm. It indicates perseverance, foresight, order and conventionality, which characteristics are entirely absent as you will observe from the conic type. The square handed people live by theory and convention; feel severely, and only act upon such impulses as their reason accepts or permits. They have continuity above all things. They expand to intelligence, but can not elevate themselves, they live not in idea, and believe only what they see with the eye, or can reason to their satisfaction.

The Knotty or philosophic hand. We find two distinct formations of this type. The sensualists and the idealists, the former deriving ideas from *external influences*, the latter from an *inner* consciousness. *Outside* of the army the Germans nearly all possess the ideal philosophic hand, lacking in animation, silent and monotonous. Science predominates over art. Theory more than action. In France the sensual philosophic prevails. Persons with this type of hands are fond of analysis and find a reason for every sensation. No idea is adopted unless thoroughly examined from every point. They are guided by reason and not instinct and claim great religious liberty, are scoffers of superstition, and enjoy all pleasures moderately. We now come to the Psychic or pointed hand. It is the most rare and beautiful type of all, and is usually small in comparison to the rest of the body. The palm is medium, fingers smooth, with delicately marked joints and ends of fingers long and drawn out to a decided point, the thumb is small and elegant. Some writers of romance have claimed that the Psychic hand belongs only to old aristocratic families. Although scarce, it nevertheless exists among the *lower classes*. These beautiful hands are unfitted for manual labor and are impractical. Everything in art attract them, and every emotion which moves the soul. In Asia this type is in the majority, whence springs the essentially religious and poetical spirit of the nations. There is a strong tendency to spiritualism, they live preeminently by soul, rather than heart. Milton, Swedenborg, Goethe, Victor Hugo and George Sand had Psychic hands. The highest lyrics, the greatest romances and most intense poems were given us by such hands. The last hand we shall class is the

Mixed type, and is usually a very complicated one, having a tendency to resemble all types. The intelligence which is revealed by a mixed hand is one which partakes of the nature of intelligences attached to each of the types represented, and are the lights and shades of all. Intermediary ideas, sciences of administration, commerce, etc., are the chief characteristics of these hands, also an aptitude for many pursuits and often never excelling in any one thing. The mind is more versatile than powerful, more amusing than instructive, and a thorough education wonderfully benefits this type of hand, which is thick and supple with large conic fingers and large thumb. In Heron Allen's translation of D'Arpentigny, he gives in conclusion some remarks upon the hands of women, and as I am indebted to this writer's translations for most of this article, I will quote them.

"The tendencies of each type are, among women, the same as among men, only those which belong to the square and spatulate types, are much less imperious and intense among women, by reason of the suppleness of the muscles than they are among men. The man *creates*, the woman *developes*.. Men have *principle*, women *form*. The former makes *laws*, the latter *morals*. 'The man is more true than the woman,' writes St. Martin, 'but she is better than he. The man is the *mind* of the woman, and she is the *soul* of the man.' Then again he values things with his brain, she with ner heart. Few women possess the knotty, philosophic hand, as few are gifted with the talent of combination, they have more tact than science, more quickness than conception, more imagination than judgement." Women usually range under two categories; those with large thumbs and those with small, the former more intelligent than sensitive, the latter more sensitive than intelligent. For clear sightedness and consideration marks the hand of the large thumbed woman, while those with small thumbs are not endowed with a high degree of sagacity. I regret very much that there is neither space or opportunity to illustrate the above various types of hands, but hope the reader will find the above explanations sufficiently intelligible to develope an interest in this fascinating science.—*Cincinnati Medical Journal.*

